# Emotional ratings and skin conductance response to visual, auditory and haptic stimuli

**DOI:** 10.1038/sdata.2018.120

**Published:** 2018-06-26

**Authors:** Elia Gatti, Elena Calzolari, Emanuela Maggioni, Marianna Obrist

**Affiliations:** 1 Sussex Computer Human Interaction (SCHI) Lab, School of Engineering and Informatics, University of Sussex, Brighton, UK; 2 Imperial College, London, UK

**Keywords:** Engineering, Psychology

## Abstract

The human emotional reactions to stimuli delivered by different sensory modalities is a topic of interest for many disciplines, from Human-Computer-Interaction to cognitive sciences. Different databases of stimuli eliciting emotional reaction are available, tested on a high number of participants. Interestingly, stimuli within one database are always of the same type. In other words, to date, no data was obtained and compared from distinct types of emotion-eliciting stimuli from the same participant. This makes it difficult to use different databases within the same experiment, limiting the complexity of experiments investigating emotional reactions. Moreover, whereas the stimuli and the participants’ rating to the stimuli are available, physiological reactions of participants to the emotional stimuli are often recorded but not shared. Here, we test stimuli delivered either through a visual, auditory, or haptic modality in a within participant experimental design. We provide the results of our study in the form of a MATLAB structure including basic demographics on the participants, the participant’s self-assessment of his/her emotional state, and his/her physiological reactions (i.e., skin conductance).

## Background & Summary

The study of human emotions is a fascinating and cross-disciplinary field of research. In the past 20 years, the interest on human emotions has been extended, from the realm of psychology, to other disciplines such us neuroscience^1^, product and experience design^2^, and computer science ^3,4^.

Despite different *theories of emotions* have been proposed over the years^5,6^, there seems to be the common understanding that emotional states are characterized by physiological and cognitive responses to clearly identifiable stimuli^7^. As such, whether we investigate emotions to understand the human mind or to teach an automated system how to be more “humane”, emotional investigation is, in the great majority of cases based on: (i) delivering emotional stimulation and (ii) measuring cognitive and physiological reactions.

With emotional stimulation, we intend an external stimulus that is able to elicit an emotional reaction. In literature, the most common way to elicit emotions is by delivering visual stimuli^8^. The International Affective Picture system (IAPS)^9^ and the Geneva Affective Pictures Database (GAPD)^10^, are examples of collection of visual stimuli (pictures) often used to elicit emotions. The Affective Norms for English Words (ANEW)^11^ and the Affective Norms for English Texts (ANET)^12^ are still visual-based (although not pictorial) collections of emotion-eliciting stimuli.

Auditory stimuli are often used as an alternative to visual stimuli (e.g., the International Affective Digital Sounds, IADS)^13^. While the IADS includes mainly short audio clips, long segments of musical pieces have also been used to elicit emotional reactions^14,15^.

It is important to note that, while IADS stimuli always have a well-defined semantic (indeed, it is not hard to imagine the source of the IADS stimuli when listening to them), classical music pieces differ from these in the sense that their emotional value is to be found within the features of the composition itself, such as its tempo and tonality^16,17^. Audio-visual stimulation has also been used to elicit emotions. A number of studies has been performed using short movies as emotional stimuli^18^ and a few standardized databases of emotional films and clips are now available^19,20^.

Alongside the more common audio-visual stimuli, more non-conventional and less standardized stimuli have been used in literature, such as olfactory^21,22^ and haptic^23–25^ stimuli.

Whatever stimulus is used to elicit an emotion, this is supposed to impact on participants’ cognitive and physiological state. To measure the impact of an emotional stimulus, a number of self-assessment questionnaires have been used in literature. Notable examples are the Self-Assessment Manikin (SAM)^26^ and the Geneva Emotion Wheel (GEW)^27^.

Together with self-assessment measures, participants’ physiological and bodily reactions to emotional stimuli are often recorded^28^. In neuroscience, such exploration is often performed through brain imaging^29^. Within computer science, where gaining access to brain activations might be cumbersome, attention is often directed towards autonomic system responses. This approach, although rather generic, allows to record bodily variations based on emotional stimulation using relatively little hardware (measuring tools)^30,31^. Such responses include the recording of parameters related with the human vascular system (blood volume pulse, heart rate), participants’ reaction times to startle reflexes, variation in skin and body temperature, and variation in the skin conductance (SC) of light electric current. The latter measure directly correlates with autonomic sudomotor nerve activation, and is therefore an indirect measure of palm sweating, in turn related with the increase in the arousal of a subject. SC is arguably the most used physiological parameter to investigate participants’ emotional activation^32,33^.

Here, we describe a database (Data Citation 1) reporting SC activations and SAM self-assessment from 100 participants, to a variety of emotional stimuli. The emotional stimuli are communicated through three different sensory modalities, namely: 20 audio stimuli, 20 visual stimuli, and 10 haptic stimuli. Of the 20 audio stimuli, 10 were selected from the IADS, and the other 10 stimuli were instrumental musical pieces that were never tested before. Similarly, 10 visual stimuli came from the IAPS, while the other 10 were abstract works of art. The 10 haptic stimuli were obtained from previous work on using mid-air haptic stimulation for eliciting emotions^25^. Noteworthy, the choice of including abstract visual stimuli was motivated by the fact that, in both the haptic and audio modality, it is possible to elicit emotions by stimuli with no obvious semantics^34^. We therefore tested whether this holds true for the selected stimuli in the visual modality.

This database is the first database that allows to compare directly SAM ratings for given stimuli to SC responses in 3 different sensory modalities. Far from being a comprehensive database of emotional stimuli, nevertheless it offers the possibility to compare ratings and physiological activation within subjects for stimuli in 3 different sensory modalities. In addition, it validates 30 stimuli, which do not have immediate meaning for the participant, for the first time assessing emotional “abstract” stimulation on a large number of participants.

## Methods

### Participants

One hundred, healthy volunteers participated in the experiment (mean=26.88 years; SD=9.11; range: from 18 to 71 years; 61 females). All but 9 participants declared to be right-handed. Pre-screening allowed only participants with normal (or corrected to normal) vision, with no history of neurological, psychological, or psychiatric disorders, and no tactile or auditory impairments to take part in the experiment. Participants were recruited from the University of Sussex, were naïve as to the purpose of the experiment, were paid for their participation, and gave written informed consent. The study was conducted in accordance with the principles of the Declaration of Helsinki and was approved by the University of Sussex ethics committee (Sciences & Technology Cross-Schools Research Ethics Committee, Ref: ER/EG296/1).

### Acquisition setup and procedure

**Figure 1:**
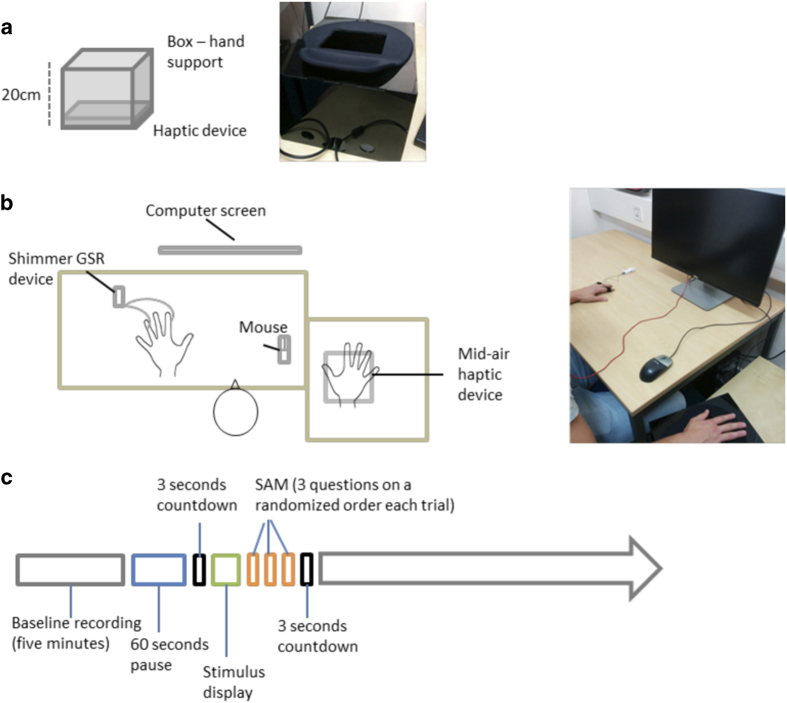
Experimental design and protocol. (**a**) Schematic representation (left) and picture (right) of the box containing the mid-air haptic device. (**b**) Schematic representation (left) and picture (right) of the experimental setup. (**c**) Schematic representation of the experimental procedure. At first the participant is asked to relax for about 5 min; followed by a 60-seconds break before the start of the experiment; a three seconds countdown precedes the stimulus; the stimulus is displayed; SAM questions are presented one after another in a randomized order; after answering to the questions the countdown for the next stimulus starts. The whole procedure is repeated through the experiment.

Participants were invited to sit comfortably in front of a computer screen. We placed SC recording electrodes (GSR Electrodes, Shimmer®) to the index and ring finger of participants’ left hand (as in ref. 35) as shown in Fig. 1.

A variable amount of time (approximately five minutes) was allowed to participants to relax, get ready for the experiment, and familiarize themselves with the experimental setup. Particularly, within this time, participants were first shown by the experimenter how to place correctly their right hand over the haptic device, and then asked to repeatedly put their right hand on it, memorizing the position of the hand so that they could replicate it throughout the experiment. Moreover, participants were invited to find a comfortable position for the left hand, which they were instructed not to move for the duration of the experiment.

At the beginning of the experiment, participants were asked again to relax for a duration of 60 s, for the SC signal to reach baseline. At the start of the experiment, SC recording was triggered, and kept running until the end of the experiment. The delivery of each emotional stimulus was marked as emotional trigger in the data log to help us interpret the meaning of the SC signal.

Emotional stimuli were presented in a randomized order. Particularly, stimuli presentation was completely randomized, rather than block randomized, to avoid any sensory-modality-related scale bias (i.e., participants adopting different scales depending on the sensory modality). Before each stimulus, a three seconds countdown appeared in the centre of the screen, followed by the stimulus. When a haptic or auditory stimulus was presented, a sentence was simultaneously displayed on the screen (either “playing audio” or “playing haptics”), informing participants on the sensory modality of the upcoming stimulus. After each stimulus, participants were asked to rate the stimulus using the original version of the SAM^26^ (see Self-Assessment rating, below), therefore rating the stimuli according to their arousal, valence, and dominance^26^, with the right hand. Each dimension of the SAM was presented in a randomized order, one after another. After answering to the last of the SAM questions, a new countdown started, marking the beginning of a new trial. At the beginning of each countdown, participants positioned their right hand comfortably on a black Plexiglas box containing a mid-air haptic device (Ultrahaptics®), as they had previously learned during the familiarization. The box was open on the upper side, and a soft support for the wrist was attached on the edge close to the participant, so that participants could comfortably place their right hand over the aperture, with the palm completely exposed to the mid-air haptic device at a 20cm distance (Fig. 1). Noteworthy, the design of the box allowed participants to easily position their hand above the device in a standardized manner. Moreover, the experimenter assisted the participant throughout the experiment and repeated the trials in case of wrong positioning of the hand.

### Stimuli

#### Auditory stimuli

The auditory stimuli comprise ten sounds from the IADS database (see Table 1), and ten instrumental extracts from various compositions (see Table 2). The ten sounds from the IADS were selected according to their SAM scores on valence and arousal reported in previous works^13^. IADS sounds were selected so that two sounds had high arousal and high valence ratings, two had low arousal and low valence ratings, two had low arousal and high valence ratings, two had high arousal and low valence ratings, and two were defined as neutral (low arousal and mid-valence ratings). The ten instrumental sounds were rated for the first time in this work, and constitute an original contribution to the state of the art. Musical pieces were considered an “abstract” form of stimulation in our study. In fact, instrumental pieces do not convey immediate meaning to the listeners, and the emotional content of the piece is instead related to features within the composition (e.g., tonality, tempo, etc.)^16,17^. The inclusion of classical music pieces in our database was motivated by the recent interest in the link between musical pieces and emotional reactions^14–17^. All the auditory stimuli were presented to participants by means of a pair of headphones (Beat Studio, Monster); auditory stimuli volume did not surpass the 90 dB limit (IADS #275, scream). The duration of all the IADS auditory stimuli selected was 6 s, apart from one lasting 5 s (see Table 1). The duration of the abstract-classic auditory stimuli could vary according to the musical sentence (see Table 1).

#### Visual stimuli

The twenty visual stimuli comprise 10 pictures from the IAPS database, and ten abstract pictures (see Table 1). The ten pictures from the IAPS were selected according to their SAM scores on valence and arousal reported in previous works^3^. In particular, two pictures were selected having high arousal and high valence ratings, two having low arousal and low valence ratings, two having low arousal and high valence ratings, and two having high arousal and low valence ratings, and two were pictures defined as neutral (low arousal and mid-valence ratings). The ten abstract pictures were arbitrarily selected from the work of renowned artists, thus their arousal, valence and dominance scores were assessed for the first time in the current work. The choice of abstract art as emotional stimulus was inspired by the body of literature on emotions, art, and aesthetic^8,36^. By including works of abstract art in our experiment, we test emotional visual stimuli that, as for the instrumental pieces in the acoustic realm, do not relate to an obvious meaning. Data reported herby might serve to further explore the connection between aesthetic and emotional reactions. All the visual stimuli were presented in the centre of a monitor screen (26 inches), placed at about 40 cm distance from participants, with the centre aligned at participants’ eye level. The visual stimuli were displayed for 15 s.

#### Haptic stimuli

Ten haptic stimuli were delivered by means of a mid-air haptic device developed by Ultrahaptics Ltd (http://ultrahaptics.com/). This mid-air haptic technology allows the creation of tactile sensations through focused ultrasound waves, resulting in sensations that can be described as dry rain, puffs of air^37^. The haptic device comprises an array of ultrasonic 16×16 transducers; each transducer can be activated individually, in sequence, or simultaneously with other transducers, thus creating unique patterns varying in location, intensity, frequency, and duration^38^.The ten different haptic stimuli used in the present experiment are presented in form of haptic patterns. These patterns were selected from a list of haptic patterns created and previously validated by Obrist and colleagues^25^. These patterns vary in location (16 different locations specified in a 4×4 grid) on the users’ palm, intensity (three level: low – medium – high), frequency (five options, range: 16–256 Hz), and duration (200–600 ms). Please note that such patterns were designed for the right hand of the user, and therefore were delivered to the right hand of the participant, without considering the hand dominance.

### Self-Assessment rating

**Figure 2:**
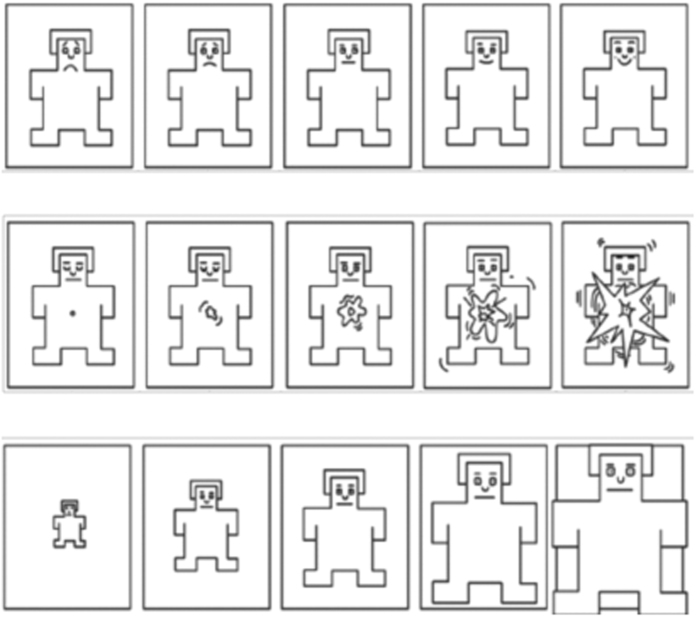
The Self-Assessment Manikin (SAM). SAM scale used in the experiment to capture participants’ emotional reactions on three dimensions: (from the top row to the bottom row) the manikin representations to express values of Valence (top), Arousal (mid), and Dominance (bottom).

Participants ratings of their own emotional reaction were recorded using the Self-Assessment Manikin (SAM) (see Fig. 2), a non-verbal pictorial assessment technique evaluating emotional reaction along three components: Valence (whether the elicited emotion is positive or negative), Arousal (how much the elicited emotion is “activating”), and Dominance (if the participant feels “in control” of the emotion), which are often identified as the main descriptors of all emotional activations^26^. The SAM was first proposed by Bradley and Lang ^24^, and reflects the idea that emotions as we know them (i.e., fear, joy, anger, calm, etc.) can be represented on a two-dimensional space that has Valence and Arousal as main orthogonal axes. Despite discussing the different theories of emotions is beyond the scope of this paper, it is worth considering the advantage of using the SAM approach as compared to a questionnaire reflecting a categorical model of emotions (i.e., Geneva Emotions Wheel). In fact, compared to a categorical approach of emotions^27^, this approach allowed us to bypass the semantic implication and idiosyncrasies that participants could have had in naming the emotion they were feeling^39^, focusing instead on the assessment of their own emotional state. Rating scales were displayed on the computer screen. Below each rating scale a horizontal bar of the same length of the five SAM’s pictorial representations was presented with a cursor at the centre of the bar. Participants had the five different manikins as a visual reference for each emotional dimension (i.e., arousal, valence, and dominance). Participants could regulate the cursor position over the bar by means of a mouse manoeuvred with their right hand. The rating scales range from 0 to 100, in 1 point steps, where 0 corresponds to the extreme left manikin, and 100 to the extreme right manikin^26^. A continuous visual analogue scale was used to account for more accuracy in the parametric data-analysis and more sensitiveness to change in the assessment^40^. No time limit was given to the participants to answer the SAM.

### Skin conductance recording and features extraction

Skin conductance (SC) response was measured with a Shimmer3 GSR+ Unit wireless device (Shimmer Sensing, Dublin). Two 8mm snap style finger TYPE (such as: Ag–AgCl) electrodes (GSR electrodes, Shimmer Sensing) with a constant voltage (0.5 V) were attached to participants’ intermediate phalanges of their left index and ring fingers. The SC recording device was connected wireless to a PC to digitalize data through the ShimmerCapture software. The gain parameter was set at 10 mSiemens (μS)/ Volt, the A/D resolution was 12 bit, allowing to record responses ranging from 2 to 100 μS.

Each recording was analysed using the MATLAB Ledalab toolbox. As first step data were downsampled and cleaned from artefacts (see *Technical validation*). Feature extraction was obtained via continuous deconvolution analysis^41^ (CDA). CDA divides the SC signal in a phasic and a tonic component, making it easier to extract features related to determinate events (triggers). Event related features obtained with CDA are shown in Table 2, which has been adapted from the original table presented at www.ledalab.de. Please note that while the trigger eliciting the event-related features was set at the beginning of the stimulus, the time window considered to compute event related features encompassed the whole duration of the stimulus, plus 4 s (for a discussion on the duration of this time window please refer to *Usage Notes and Limitations of the database,* below). Standard trough-to-peak (TTP) features were also obtained from the raw signal, as well as global measures (mean and maximum deflection, see Table 2).

**Figure 3:**
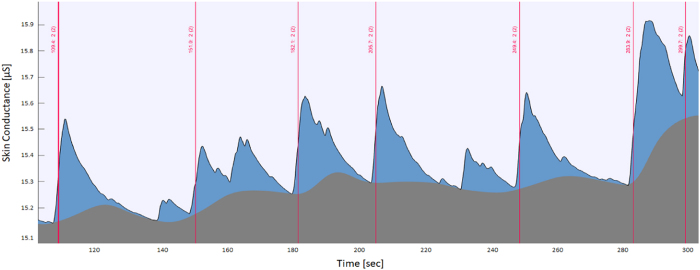
Example of a Skin Conductance (SC) recording as displayed from the analysis software Ledalab. The blue area indicates the phasic component of the signal, while the grey area represents the tonic component. The red line indicates the trigger (moment of delivery of the stimulus).

In general, features related to SC response have been related in multiple occasions to emotional responses (and particularly, high arousal responses)^31–33^. Emotional states characterized by high arousal correlate with the activation of a fight or fly response. Such response is regulated by the autonomic system (and in particular by the sympathetic system), which in turn activates the sudomotor nerve, triggering the release of sweat from the sweat glands in the hand’s skin. An example of SC signal is shown in Fig. 3.

## Data Records

SC recordings and self-assessment ratings collected during the experiment are organized in a single MATLAB data structure available at (Data Citation 1). Such data structure also includes demographic information on each participant, as well as baseline SC recordings collected prior the experiment. Quality of the SC recording is also available as further structure field (see Technical Validation). Moreover, the “features” field of the structures shows SC features extracted by using Continuous Decomposition Analysis (CDA) with the MATLAB toolbox Ledalab.

### multisensory_emotions.m

**Figure 4:**
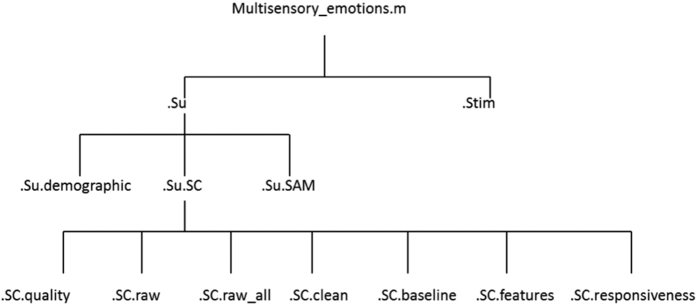
Graphical representation of the MATLAB structure containing all data from each participant.

Hereafter is an explanation of all the fields of the MATLAB data structure multisensory_emotions.m are explained. Matlab structures allow to organize the data hierarchically, the hierarchical design of the structure is shown in Fig. 4.

- *multisensory_emotions.stim=*50X6 MATLAB array. This field allows to retrieve information about the stimuli that were delivered to participants during the experiment. *multisensory_emotions.stim* allows users to access a MATLAB array which columns represent respectively: the stimulus identification number, the modality of delivery (either audio, video, or haptic), the name of the stimulus (IAPS/IADS identification number, artwork title, etc.), the duration of the stimulus/presentation time, the author/composer (when known), and finally the database on which the stimulus was tested (in case of further expansion of the present database by other parties in future studies, see below: *Usage Notes and Limitations of the database*).
- *multisensory_emotions.su*=The.*su* field allows to retrieve information about one particular participant. The.*su* field is the basic layer of the structure. The number of items at this level of the structure is 100, same as the number of participants taking part to the experiment. The*.su* field is, in turn, divided into different sub-fields:
- *.su.demographic =*1X4 MATLAB vector. Accessing to the*.demographic* field allows to retrieve demographic information on one particular participant. The vector contains, respectively: the participant’s ID number, the gender of the participant, the hand dominance, and the age of the participant.
- *.su.SAM=*MATLAB array 50X5. Accessing the*.SAM* field allows to retrieve information about the ratings of each stimulus, as well as about the order of the stimuli presented to the participant..array columns are, respectively, ratings on (1) valence, (2) arousal, and (3) dominance. Column number 4 represents the stimulus identification number. It is possible look at the type of stimulus by matching the identification number with the stimulus identification number in *multisensory_emotions.stim.* Please note that the knowledge of what stimulus was delivered to the participant in any given moment is a crucial information to correctly interpret the SC signals. Column number 5 marks instead the presentation order of the stimuli.
- *.su.SC=*the*.SC* field allows users to access skin conductance data collected during the experiment. SC data is in turn divided into 7 sub fields:
- *.SC.raw:* nX2 MATLAB array containing the n samples collected throughout the whole experiment and 2 columns. The first column represents skin conductance value (μS) collected with the Shimmer3 GSR+ Unit. The second column represents the presentation of the stimulus as SC trigger. The moment of the stimulus presentation is marked as 1, while other samples are simply 0 s.
- *.SC.raw_all:* nX2 MATLAB array containing the n samples collected throughout the whole experiment and 2 columns. The first column represents skin conductance value (μS) collected with the Shimmer3 GSR+ Unit. The second column represents the presentation of the stimulus as SC trigger. The moment of the stimulus presentation is marked as 1 and the presentation of each SAM question on arousal is marked as 4, on valence as 5, on dominance as 6.
- *.SC.clean:* nx2 MATLAB matrix containing the n samples collected throughout the whole experiment and 2 columns. The data in*.SC.clean* have been downsampled with a factor 4 to allow for a faster reading and elaboration of the SC trace. Moreover, data in this sub-field have been cleaned using the Ledalab artefact correction toolbox (see Technical Validation below).
- *.SC.baseline:* 1xn MATLAB vector, including the SC recordings from the participant prior the beginning of the experiment.
- *.SC.quality:* single value from 0 to 2, whereas 0 represents unusable data, 1 represents partially complete recording, and 2 represents complete recordings.
- *.SC.features:* 50X12 MATLAB array. The columns represent the 12 features extracted using the Ledalab toolbox for MATLAB (see Technical Validation for more information on the features extracted). The rows represent the stimuli arranged by “stimulus number” (therefore not presentation order). Missing values are reported as NA in the MATLAB table. Each of the 12 features is reported and explained in Table 2.
- *.SC.responsiveness:* single value, either 0 or 1. It is meant to serve as an indication of whether the participant showed any phasic response throughout the experiment. The absence of phasic responses for all the stimuli *(.SCrespnsiveness*=0) mark the participant as a possible non-responder. With non-responder is meant an individual with low skin conductance, on which SC recording do not evidence emotional responses even when they are present (as shown by SAM ratings).

Together with the MATLAB structure, we also provide an R list (multisensory_emotions_R.rda, Data Citation 1). The R list architecture is equivalent to the one shown in Fig. 4. R is an open source software downloadable at https://cran.r-project.org/ and hence enables anyone interested in our dataset to access and use it for future studies.

## Technical Validation

A post-doctoral level researcher with over 5 years of experience in the field of behavioural research acquired data from all the 100 participants. Participants were invited to take a comfortable position at the beginning of the experiment, and asked not to move their left hand during the whole experiment to avoid movement artefacts in the SC signal.

### Participants reliability and SAM ratings

While for the SC signal, it is possible to assess the quality of the data by checking the number of obtained samples and artefacts, it can be harder to determine whether self-assessment questionnaires have been answered by participants with the due attention. To investigate the reliability of our data, we compared the ratings obtained in the 10 IAPS and 10 IADS stimuli in our experiment with the values of valence, arousal, and dominance indicated in the IAPS and IADS manuals^9,13^. Results showed high significant correlations between our data and the expected value from the two manuals (IADS: r=0.86, p<0.01; IAPS: r=0.79, p<0.01). We also checked whether the responses distribution for each stimulus and each SAM dimension approximated normality. Table 3 (available online only) shows average ratings±standard deviation for each stimulus in the arousal, valence, and dominance dimensions. Table 4 (available online only) additionally shows results from a normality test (Kolmogorov–Smirnov test) and the skewness and kurtosis of their distributions.

### Skin conductance signal processing

From each participant, we obtained two SC traces. The first trace was collected in absence of stimulation for about 60 s *(.SC.baseline)*. The second trace was collected for the whole duration of the experiment.

**Figure 5:**
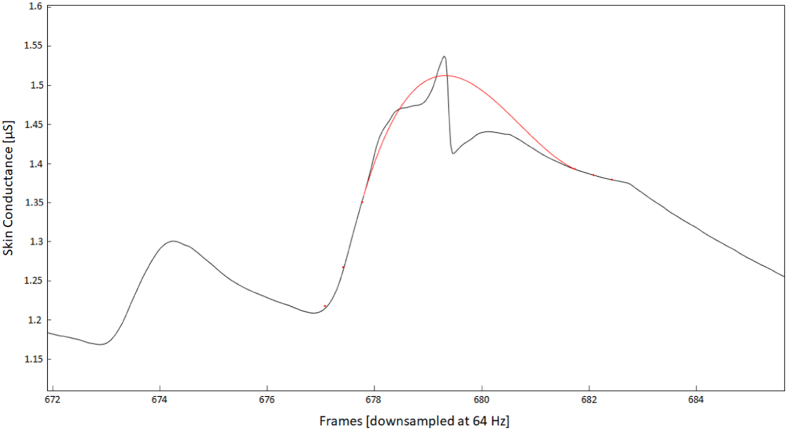
Example of spline fitting to correct a signal artefact. The SC signal (black trace) spikes are likely a result of the movement of the participant. The spike is identified through thorough visual inspection and a spline (red line) is fitted to the data to exclude the artefact and recover the signal.

Both traces were examined by using the Ledalab MATLAB toolbox. Ledalab allows to visually inspect each single loaded trace and correct for movement artefacts. Before artefact correction, signal was downsampled (with a factor 8) to allow for a faster processing of the data. The movement artefacts were identified after careful visual inspection, and corrected by using a fitting spline. An example of such correction can be seen in Fig. 5.

SC signal ranged between 10.01 μS and 28.34 μS (mean: 15.55, sd: 1.67, value computed on SC traces rated one or more, see SC.quality). Collected SC values are in line with typical SC levels in humans, with baseline SC usually lower than the SC recording during the experiment^29^. Downsampled and artefacts corrected data are available at.*SC.clean.* Raw data at full sampling (512 Hz) are available at*.SC.raw.*

### Skin conductance signal processing

Skin conductance signal is known to change in time ^31,32^. Particularly, the tonic component of the signal (Fig. 3) is expected to vary^41^ over relatively long periods of time. On the contrary, the phasic component of the signal, if correctly extracted from the raw data, is expected only to be tied to the eliciting events (that is: the emotional stimulus). However, it is important to consider that the effect of the triggering event on the SC signal might also decrease after several presentations of emotional stimuli, as the participant could habituate to the emotional stimulation itself. In other words, by being emotionally stimulated repeatedly, a participant could grow accustomed to the emotional impact of the eliciting stimuli, therefore not being scared, moved, or aroused by a stimulus as it would be if the stimulus was presented at the beginning of the experiment. We used Spearman rank-order correlation to assess whether the position that a stimulus held in the sequence of trials (that is, whether the stimulus was presented as first, second, third, etc.) correlated with lower (or higher) value of particular phasic features. Results are shown in Table 5. As it is possible to evince by the results in the table, correlations were low but nonetheless present, and users will have to take this fact into account when using the database.

## Usage Notes and Limitations of the database

The proposed database is available at (Data Citation 1) and can be used for several different applications. These data are of clear interest for different fields investigating human emotional reaction and automatic emotion recognition, from psychology to computer science. We strongly encourage the use of the stimuli contained in this database for experiments where emotional stimulation is needed, and particularly when emotional stimulation is not delivered through “traditional” means (such as pictures). In particular, the emotional ratings and reactions to haptic stimuli constitute a first, however small, strongly validated database for affective haptic. Abstract art pieces and instrumental pieces are also not conventional and validated stimuli. At the same time, SC traces and features could be used to train automatic systems in recognizing human emotions, given the sensory modality through which the stimulus has been delivered. Furthermore, the relationship between SAM ratings, SC features, and sensory modality involved in the stimulation could open new paths for the study of how emotions are communicated and interpreted by humans.

In order to facilitate the access to the data within the database, a MATLAB function and its equivalent R function (*SCR_statistics.m* and *SCRstatistics.R*) have been prepared. These functions are available at (Data Citation 1). These functions allow to compute mean and standard deviation over the selected SCR features across all participants, for each different stimulus. Furthermore, these functions allow the user to select the participants over which compute the descriptive statistics based on the quality of their SC.

However, we would also point out some of the flaws of the current database, so to help the future users in interpreting the results that are available for its analysis. First, this database is far from being complete and comprehensive of all the possible stimuli available in the three different sensory modalities. The number of conditions tested strongly reduced the number of samples we could use, as we wanted to maintain an experiment of a reasonable duration. Any generalization of the data contained in this database should therefore be limited at the stimuli hereby tested. Importantly, we encourage other scientists to expand the database themselves, still maintaining the same experimental design, where multisensory stimuli for eliciting emotions are delivered to users in a within-subjects experimental design. To facilitate the inclusion of further stimuli and participants responses to the database, we developed an R-based graphic user interface which allows other researchers to merge their new data to the R list. The graphic user interface is available at [Data Citation 1] and has been developed using the open source R package shiny. Such interface provides information about the format required to integrate the data and allows a database to be included by following a series of guided steps, even when the SC or the SAM fields are missing.

Second, SCR were computed over different timeframes. Indeed, the response window in which SCRs were considered depend on the duration of the stimulus. The choice was motivated by the fact that for stimuli such as haptic and auditory (abstract) any truncation of the stimulus would have resulted in an uncomplete pattern, or melody, therefore (most likely) inducing frustration in the participant. Finally, our last concern relates to the haptic stimuli used. Although the positioning of the hand was relatively constant across trials, and facilitated by the foam support on top of the haptic device, the size of the hand was different across participants. This could lead the haptic stimuli to fall on slightly different locations on the hand depending on the participant. Therefore, haptic stimulation may not have been identical across participants, whereas audio and visual stimulation was.

## Additional information

**How to cite this article**: Gatti, E, *et al.* Emotional ratings and skin conductance response to visual, auditory and haptic stimuli. *Sci. Data* 5:180120 doi: 10.1038/sdata.2018.120 (2018).

**Publisher**’**s note**: Springer Nature remains neutral with regard to jurisdictional claims in published maps and institutional affiliations.

## Supplementary Material



## Figures and Tables

**Table 1:** Multisensory stimuli used in the experiment.

Stimulus number	Stimulus type	Stimulus name	Stimulus author	Display duration
1	AUDIO CLASSIC	Piano Concerto No. 20 in D minor, K466	W.A. Mozart	13
2	AUDIO CLASSIC	Olympic fanfare and theme	J. Williams	19
3	AUDIO CLASSIC	October	E. Whitacre	38
4	AUDIO CLASSIC	Shenandoah	F. Ticheli	45
5	AUDIO CLASSIC	Danse macabre	C. Saint-Saëns	13
6	AUDIO CLASSIC	Night on the bald mountain	M.P. Mussorgsky	30
7	AUDIO CLASSIC	Sweet death	J.S. Bach	41
8	AUDIO CLASSIC	Adagio for Strings	S. Barber	30
9	AUDIO CLASSIC	Undertale Extended – Undertale OST	T. Fox	39
10	AUDIO CLASSIC	Megalovania Extended – Undertale OST	T.Fox	30
11	AUDIO IADS	#110 baby laughing	NA	6
12	AUDIO IADS	#115 bees	NA	6
13	AUDIO IADS	#172 water	NA	6
14	AUDIO IADS	#230 women laughing	NA	6
15	AUDIO IADS	#255 vomiting	NA	6
16	AUDIO IADS	#260 babies crying	NA	6
17	AUDIO IADS	#275 scream	NA	5
18	AUDIO IADS	#320 typewriter	NA	6
19	AUDIO IADS	#351 clapping	NA	6
20	AUDIO IADS	#364 crowd voices party	NA	6
21	PICTURES ABSTRACT	La vague	H. Matisse	15
22	PICTURES ABSTRACT	Black Square	K. S. Malevich	15
23	PICTURES ABSTRACT	Rythme n1	R. Delaunay	15
24	PICTURES ABSTRACT	Lines areas depth III	F. Kupka	15
25	PICTURES ABSTRACT	1866-1944 Ohne Titel	V. V. Kandinsky	15
26	PICTURES ABSTRACT	Mondrian	P. Mondrian	15
27	PICTURES ABSTRACT	Based on Leaf Forms and Spaces	Dove	15
28	PICTURES ABSTRACT	Fire in the Evening	P. Klee	15
29	PICTURES ABSTRACT	Udnie	F. Picabia	15
30	PICTURES ABSTRACT	Yellow Red Blue	V. V. Kandinsky	15
31	PICTURES IAPS	#1274 beetles	NA	15
32	PICTURES IAPS	#1525 dog growling	NA	15
33	PICTURES IAPS	#2045 baby smiling1	NA	15
34	PICTURES IAPS	#2070 baby smiling2(female)	NA	15
35	PICTURES IAPS	#2900 kid crying	NA	15
36	PICTURES IAPS	#3550 man in blood	NA	15
37	PICTURES IAPS	#5829 sunset	NA	15
38	PICTURES IAPS	#7009 empty mug	NA	15
39	PICTURES IAPS	#7238 blue and yellow spheres	NA	15
40	PICTURES IAPS	#8470 athlete exults	NA	15
41	HAPTIC^25^	P1	NA	1.4
42	HAPTIC^25^	P2	NA	2.03
43	HAPTIC^23^	P3	NA	1.3
44	HAPTIC^25^	P4	NA	1.2
45	HAPTIC^25^	P5	NA	1.4
46	HAPTIC^25^	P6	NA	1.2
47	HAPTIC^25^	P7	NA	1.2
48	HAPTIC^25^	P8	NA	1.3
49	HAPTIC^25^	P9	NA	1.2
50	HAPTIC^25^	P10	NA	1.4

**Table 2:** SC signal’s features, available at*.SC.features*

Variable (Labels used in exported files)	Description
Event data	
Event.nr	Sequence number of event/marker
Event.nid	Numerical ID of event
event.name	Optional name or description of event
Event.ud	Optional user data associated with event
*Continuous Decomposition Analysis (CDA) (Extraction of Continuous Phasic/Tonic Activity based on Standard Deconvolution)*	
CDA.nSCR	Number of significant^a^ SCRs (Skin Conductance Responses)
CDA.Latency	Response latency of first significant^a^ SCR within response window b[s].
CDA.AmpSum	Sum of significant^a^ SCR-amplitudes of SCRs within response window^b^. (reconvolved from corresponding phasic driver-peaks) [μS]
CDA.SCR	Average phasic driver within response window^b^. This score represents phasic activity within response window^b^ most accurately, but does not fall back on classic SCR amplitudes [μS]
CDA.ISCR	Area (i.e. time integral) of phasic driver within response window. It equals SCR multiplied by size of response window b[μS^b^s]
CDA.PhasicMax	Maximum value of phasic activity within response window^b^ [μS]
CDA.Tonic	Mean tonic activity within response window^b^ (of decomposed tonic component)
*Standard trough-to-peak (TTP) or min-max analysis*	
TTP.nSCR	Number of significant^a^ SCRs, within response window^b^
TTP.AmpSum	Sum of SCR-amplitudes of significant^a^ SCRs within response window^b^ [μS]
TTP.Latency	Response latency of first significant^a^ SCR within response window^b^ [s]
*Global Measures*	
Global.Mean	Mean SC value within response window^b^ (within response window)
Global.MaxDeflection	Maximum positive deflection within response window^b^

^a^significant=which amplitude surpasses the threshold of 0.01 μS^41^

^b^within response window=the response window considered for each stimulus was different, and was equal to the whole duration of the stimulus, plus additional 300 ms to account for the relatively slow dynamics of SC response.

**Table 3:** SAM results for each stimulus averaged across participants-

Stimulus number	Arousal Mean	Standard deviation	Valence Mean	Standard deviation	Dominance Mean	Standard deviation
**1**	67.75±	21.24	80.63±	11.85	57.75±	25.97
**2**	69.19±	22.46	76.94±	22.46	54.68±	22.46
**3**	53.7±	24.53	71.14±	24.53	57.77±	24.53
**4**	58.32±	24.39	69.17±	24.39	55.69±	24.39
**5**	56.48±	21.55	57.94±	21.55	59.9±	21.55
**6**	72.41±	17.49	49.81±	17.49	45.95±	17.49
**7**	45.32±	25.08	45.76±	25.08	53.81±	25.08
**8**	57.37±	24.77	45.7±	24.77	51.76±	24.77
**9**	50.51±	22.38	67.44±	22.38	66.22±	22.38
**10**	72.89±	22.95	70.02±	22.95	54.05±	22.95
**11**	64.31±	21.74	77.41±	21.74	57.84±	21.74
**12**	61.85±	25.61	26.8±	25.61	46.75±	25.61
**13**	47.8±	23.22	71±	23.22	64.25±	23.22
**14**	50.05±	20.46	64. 06±	20.46	63.54±	20.46
**15**	67.97±	23.81	18.54±	23.81	37.89±	23.81
**16**	64.56±	21.5	29.34±	21.5	43.7±	21.5
**17**	78.07±	24.17	14.39±	24.17	28.89±	24.17
**18**	51.54±	22.85	40.59±	22.85	62.16±	22.85
**19**	54.95±	22.3	69.41±	22.3	63.39±	22.3
**20**	59.67±	20.27	55.47±	20.27	59.22±	20.27
**21**	31.78±	22.8	56.66±	22.8	71.77±	22.8
**22**	30.35±	23.22	47.25±	23.22	71.14±	23.22
**23**	47.14±	24.73	61.52±	24.73	69.48±	24.73
**24**	37.56±	22.07	50.07±	22.07	68.47±	22.07
**25**	47.35±	21.69	60.96±	21.69	66.35±	21.69
**26**	36.96±	24.4	53.89±	24.4	71.05±	24.4
**27**	36.33±	23.56	48.9±	23.56	70.22±	23.56
**28**	37.26±	23.12	52.92±	23.12	68.72±	23.12
**29**	40.59±	22.88	51.37±	22.88	69.69±	22.88
**30**	47.39±	26.4	64.25±	26.4	69.58±	26.4
**31**	56.74±	26.44	25.84±	26.44	47.26±	26.44
**32**	62.75±	23.71	24.91±	23.71	43.21±	23.71
**33**	51.52±	23.5	75.13±	23.5	65±	23.5
**34**	53.75±	22.46	77.73±	22.46	59.73±	22.46
**35**	56.23±	21.66	23.91±	21.66	50.52±	21.66
**36**	63.79±	22.53	19.7±	22.53	47.29±	22.53
**37**	50.43±	26.12	78.89±	26.12	65.42±	26.12
**38**	26.1±	22.79	54.37±	22.79	79.36±	22.79
**39**	44.18±	24.15	57.49±	24.15	68.67±	24.15
**40**	50.44±	21.99	72.41±	21.99	69.53±	21.99
**41**	52.51±	21.03	65.94±	21.03	58.28±	21.03
**42**	44.94±	21.5	57.38±	21.5	62.51±	21.5
**43**	44.77±	23.61	60.28±	23.61	62.87±	23.61
**44**	55.24±	22.85	62.25±	22.85	56.74±	22.85
**45**	38.3±	23.28	54.6±	23.28	66.08±	23.28
**46**	44.93±	22.78	59.2±	22.78	62.69±	22.78
**47**	41.31±	23.17	54.81±	23.17	63.74±	23.17
**48**	40.31±	21.88	56.26±	21.88	65.13±	21.88
**49**	46.74±	22.01	57.83±	22.01	63.43±	22.01
**50**	36.64±	24.14	55.74±	24.14	67.15±	24.14

**Table 4:** SAM results normality assessment-

	Kolmogorov-Smirnov normality test (p-values)			Distribution Skewness			Distribution Kurtosis		
Stimulus	Arousal	Valence	Dominance	Arousal	Valence	Dominance	Arousal	Valence	Dominance
**1**	8.14E-86	2.45E-89	1.44E-87	−1.13	−0.36	0.02	4.29	2.97	1.91
**2**	8.24E-86	2.45E-89	2.45E-89	−1.14	−0.76	0.18	4.09	3.39	1.8
**3**	8.24E-86	2.45E-89	2.45E-89	−0.28	−0.61	−0.09	2.13	3.03	1.82
**4**	8.24E-86	2.45E-89	4.26E-89	−0.5	−0.68	−0.02	2.65	2.65	1.91
**5**	1.40E-85	2.45E-89	1.44E-87	−0.59	0.24	−0.24	2.93	2.49	2.12
**6**	1.44E-87	2.45E-89	1.44E-87	−1.09	0.07	0.5	5.68	2.13	2.15
**7**	2.55E-77	1.40E-85	1.44E-87	−0.14	0.23	0.15	1.98	2.23	1.82
**8**	4.41E-84	4.26E-89	1.44E-87	−0.51	0.34	0.36	2.57	2.06	1.92
**9**	2.45E-89	2.45E-89	2.48E-89	−0.23	−0.43	−0.38	2.25	3.28	2.07
**10**	2.45E-89	1.44E-87	1.44E-87	−1.3	−0.92	−0.01	4.36	3.32	1.89
**11**	8.14E-86	2.45E-89	2.45E-89	−0.87	−0.87	−0.11	3.63	2.58	1.95
**12**	7.53E-84	1.93E-80	4.26E-89	−0.85	0.47	0.43	3.04	3.04	2.05
**13**	2.29E-82	2.45E-89	2.45E-89	−0.26	−0.21	−0.18	2.33	2.41	2.07
**14**	1.14E-80	2.45E-89	2.45E-89	−0.67	−0.15	−0.17	2.77	2.18	2.03
**15**	2.29E-82	5.60E-66	5.55E-79	−1.03	1.68	0.65	4.05	8.44	2.54
**16**	4.41E-84	1.93E-80	1.14E-80	−1.01	1.21	0.38	4.21	5.48	2.45
**17**	4.41E-84	2.31E-63	2.31E-63	−1.68	0.86	0.96	5.54	2.79	2.85
**18**	1.11E-75	1.46E-87	2.45E-89	−0.71	−0.27	−0.07	2.87	4.17	1.97
**19**	1.44E-87	2.45E-89	2.45E-89	−0.59	−0.12	−0.19	2.59	1.99	1.77
**20**	8.14E-86	1.44E-87	2.45E-89	−0.76	−0.27	0.18	3.39	2.6	1.83
**21**	3.15E-72	2.45E-89	2.45E-89	0.3	0.08	−0.63	1.93	4.1	2.35
**22**	4.56E-69	1.44E-87	1.44E-87	0.53	0.24	−0.69	2.16	5.28	2.46
**23**	2.52E-77	2.45E-89	1.44E-87	−0.48	0.37	−0.64	2.13	2.75	2.76
**24**	5.55E-79	1.44E-87	1.44E-87	−0.02	0.16	−0.46	2.13	3.64	2.47
**25**	7.53E-84	2.45E-89	1.44E-87	−0.28	0.11	−0.34	2.42	2.73	2.3
**26**	4.21E-77	1.44E-87	1.44E-87	0.16	0.19	−0.59	2.08	4.05	2.68
**27**	1.22E-70	2.45E-89	2.48E-89	0.1	0.19	−0.53	2.18	3.83	2.39
**28**	7.49E-71	2.45E-89	1.44E-87	−0.07	0.38	−0.53	1.83	3.49	2.35
**29**	9.21E-79	2.45E-89	4.26E-89	−0.03	−0.05	−0.51	1.97	2.95	2.22
**30**	4.72E-74	2.45E-89	1.44E-87	−0.23	0.26	−0.54	2.21	2.1	2.57
**31**	2.29E-82	1.85E-64	3.90E-82	−0.41	0.55	0.23	2.38	3.14	1.66
**32**	4.41E-84	1.85E-75	4.46E-84	−0.79	0.43	0.42	3.03	3.05	2.06
**33**	1.16E-80	2.45E-89	2.45E-89	−0.61	−0.34	−0.47	2.71	2.29	2.25
**34**	4.41E-84	2.45E-89	2.45E-89	−0.5	−0.75	−0.23	2.69	2.98	1.84
**35**	2.29E-82	4.72E-74	8.24E-86	−0.75	0.15	0.21	3.57	2.52	2.13
**36**	4.41E-84	1.85E-64	1.44E-87	−0.96	0.56	0.38	3.71	2.5	1.96
**37**	4.41E-84	2.45E-89	4.26E-89	−0.05	−0.92	−0.34	2.08	3.44	2.19
**38**	2.31E-63	2.45E-89	2.45E-89	0.55	1.03	−1.03	2.04	4.68	3.17
**39**	1.13E-75	2.45E-89	8.14E-86	−0.33	0.43	−0.71	2.08	2.9	2.81
**40**	8.14E-86	2.45E-89	4.26E-89	−0.35	−0.06	−0.66	2.72	1.99	2.67
**41**	8.24E-86	2.45E-89	2.45E-89	−0.6	0.17	−0.01	3.01	2.21	1.84
**42**	4.46E-84	2.45E-89	2.45E-89	−0.33	0.38	−0.04	2.43	3.69	1.75
**43**	1.58E-78	2.45E-89	2.45E-89	−0.36	0.05	−0.15	2.19	3.34	2.08
**44**	4.46E-84	2.45E-89	2.45E-89	−0.7	−0.21	0.26	2.95	2.72	1.86
**45**	9.21E-79	2.45E-89	2.45E-89	0.04	1.05	−0.17	2.02	3.96	1.62
**46**	2.32E-82	2.45E-89	2.45E-89	−0.05	0.52	−0.2	2.58	2.91	1.99
**47**	2.49E-87	2.45E-89	2.45E-89	0.06	0.63	−0.16	2.01	3.34	1.91
**48**	5.48E-79	2.45E-89	2.45E-89	0.03	0.31	−0.11	2.12	3.11	1.84
**49**	3.90E-82	2.45E-89	2.45E-89	−0.37	−0.03	−0.08	2.27	2.96	2.19
**50**	9.21E-79	1.44E-87	2.48E-89	0.39	−0.26	−0.5	2.51	4.57	2.46

**Table 5:** Spearman rank order correlations between features and the position stimuli were presented to participants.

Feature	rho	p-value(95% confidence interval)
CDA_nSCR	−0.270	<0.01
CDA_Latency	−0.107	<0.01
CDA_AmpSum	−0.214	<0.01
CDA_SCR	0.007	0.59
CDA_ISCR	−0.263	<0.01
CDA_PhasicMax	−0.114	<0.01
